# Switching warfarin to direct oral anticoagulants in atrial fibrillation: Insights from the NCDR PINNACLE registry

**DOI:** 10.1002/clc.23376

**Published:** 2020-05-06

**Authors:** Christopher T. Sciria, Thomas M. Maddox, Lucas Marzec, Benjamin Rodwin, Salim S. Virani, Amarnath Annapureddy, James V. Freeman, Ali O'Hare, Yuyin Liu, Yang Song, Gheorghe Doros, Yue Zheng, Jane J. Lee, Ramesh Daggubati, Lina Vadlamani, Christopher Cannon, Nihar R. Desai

**Affiliations:** ^1^ Division of Cardiology, New York‐Presbyterian Hospital Weill Cornell Medical Center, New York New York New York USA; ^2^ Division of Cardiology Washington University School of Medicine St. Louis Missouri USA; ^3^ Department of Cardiology Kaiser Permanente Lafayette Colorado USA; ^4^ Department of Medicine VA Connecticut Healthcare System West Haven CT USA; ^5^ Michael E. DeBakey Veterans Affairs Medical Center and Section of Cardiovascular Research Baylor College of Medicine Houston Texas USA; ^6^ Yale Center for Outcomes Research and Evaluation (CORE) New Haven Connecticut USA; ^7^ Division of Cardiovascular Medicine Yale School of Medicine New Haven Connecticut USA; ^8^ Baim Institute for Clinical Research Boston Massachusetts USA; ^9^ Division of Cardiology Winthrop University Hospital Mineola New York USA; ^10^ Yale School of Medicine New Haven Connecticut USA; ^11^ Brigham and Women's Hospital Heart & Vascular Center and Harvard Medical School Boston Massachusetts USA

**Keywords:** atrial fibrillation, direct oral anticoagulants, patterns of care, practice patterns, warfarin

## Abstract

**Background:**

Previous studies examining the use of direct oral anticoagulants (DOACs) in atrial fibrillation (AF) have largely focused on patients newly initiating therapy. Little is known about the prevalence/patterns of switching to DOACs among AF patients initially treated with warfarin.

**Hypothesis:**

To examine patterns of anticoagulation among patients chronically managed with warfarin upon the availability of DOACs and identify patient/practice‐level factors associated with switching from chronic warfarin therapy to a DOAC.

**Methods:**

Prospective cohort study of AF patients in the NCDR PINNACLE registry prescribed warfarin between May 1, 2008 and May 1, 2015. Patients were followed at least 1 year (median length of follow‐up 375 days, IQR 154‐375) through May 1, 2016 and stratified as follows: continued warfarin, switched to DOAC, or discontinued anticoagulation. To identify significant predictors of switching, a three‐level multivariable hierarchical regression was developed.

**Results:**

Among 383 008 AF patients initially prescribed warfarin, 16.3% (n = 62 620) switched to DOACs, 68.8% (n = 263 609) continued warfarin, and 14.8% (n = 56 779) discontinued anticoagulation. Among those switched, 37.6% received dabigatran, 37.0% rivaroxaban, 24.4% apixaban, and 1.0% edoxaban. Switched patients were more likely to be younger, women, white, and have private insurance (all *P* < .001). Switching was less likely with increased stroke risk (OR, 0.92; 95%CI, 0.91‐0.93 per 1‐point increase CHA_2_DS_2_‐VASc), but more likely with increased bleeding risk (OR, 1.12; 95%CI, 1.10‐1.13 per 1‐point increase HAS‐BLED). There was substantial variation at the practice‐level (MOR, 2.33; 95%CI, 2.12‐2.58) and among providers within the same practice (MOR, 1.46; 95%CI, 1.43‐1.49).

**Conclusions:**

Among AF patients treated with warfarin between October 1, 2010 and May 1, 2016, one in six were switched to DOACs, with differences across sociodemographic/clinical characteristics and substantial practice‐level variation. In the context of current guidelines which favor DOACs over warfarin, these findings help benchmark performance and identify areas of improvement.

**Abbreviations**
AF: atrial fibrillation
DOAC: direct oral anticoagulant
ACC/AHA/HRS: American College of Cardiology/American Heart Association/Heart Rhythm Society
NCDR: National Cardiovascular Disease Registry
  PINNACLE
  Practice Innovation and Clinical Excellence
CKD: chronic kidney disease
GFR: glomerular filtration rate

## 1 INTRODUCTION

Anticoagulation significantly decreases stroke risk in atrial fibrillation (AF).^1^ Although warfarin was previously the standard treatment for stroke prevention in AF, direct oral anticoagulant (DOAC) medications have been approved for nonvalvular AF since 2010. Compared to warfarin, these medications have been shown to provide more consistent anticoagulant effect, substantially reduce the risk of intracranial hemorrhage, have fewer interactions with other drugs/food, and do not require international normalized ratio (INR) monitoring.^2^, ^3^ As a result, the 2019 update to the American College of Cardiology (ACC)/American Heart Association (AHA)/Heart Rhythm Society (HRS) AF guidelines favor the use of DOACs over warfarin for stroke prevention in nonvalvular AF with a class 1A recommendation.^4^

Previous studies from the National Cardiovascular Disease Registry's (NCDR) Practice Innovation and Clinical Excellence (PINNACLE) registry have examined patterns of DOAC use in AF, and have noted disparities in DOAC use based on sex, race, and insurance status.^5^, ^6^, ^7^, ^8^, ^9^ However, these analyses have focused exclusively on patients newly initiating anticoagulation for AF. There is limited data evaluating how commonly patients on warfarin are switched to DOACs and why they are switched.^7^ The NCDR PINNACLE registry longitudinally follows patients with AF, providing insight into patterns of switching oral anticoagulant therapy and identifying patient and practice‐level factors associated with switching.^10^

Accordingly, we sought to examine patterns of anticoagulation among patients with AF treated with warfarin, determine predictors of switching from warfarin to DOACs, and identify potential disparities in care based on sociodemographic and clinical factors. We hypothesized that there would be significant disparities in switching patterns and substantial patient/practice‐level variations.

## 2 METHODS

### 2.1 Data source

The NCDR PINNACLE registry is a prospective outpatient quality improvement registry that was created by the ACC in 2008.^10^ In this registry, both academic and private practices collect longitudinal data pertaining to the care of patients with AF, coronary artery disease, hypertension, and heart failure. This includes demographics, medical comorbidities, medications, and practice/provider data. These data are collected from the patients' charts using a standardized collection tool to obtain and transmit data. De‐identified data was extracted from an electronic medical record under a quality improvement model, with approval from an institutional review board and informed consent waived. Rigorous data definitions and periodic data quality audits are conducted to maintain NCDR data quality assurance.^11^ Analysis of the data was performed at the Baim Institute for Clinical Research.

### 2.2 Study population

The study cohort included all patients with nonvalvular AF, in the PINNACLE dataset with at least one warfarin prescription between January 1, 2008 and May 1, 2015. Patients were followed longitudinally in PINNACLE and stratified based upon their anticoagulation between October 1, 2010 and May 1, 2016 as follows: (a) continued warfarin, (b) switched to a DOAC, and (c) discontinued anticoagulation. Patients were substratified by the date of index visit (2010‐2011, 2012‐2013, and 2014‐2016) and time after index visit (30 days, 90 days, 180 days, 365 days, and more than 365 days). Patients were considered to have switched if they had a DOAC recorded on two consecutive visits in the registry. Patients were excluded if they were younger than 18 years of age, or did not have a second encounter after the initiation of warfarin. Additionally, patients were excluded if they had a history of cardiac valve surgery (valve replacement/valvular AF) or systemic embolism recorded in the registry, in order to analyze patients who were potentially eligible to switch.

### 2.3 Statistical methods

The primary outcome of this study was the proportion of patients with AF treated with warfarin that were switched to a DOAC and the proportion switched to each specific agent. We calculated the proportion of patients with AF treated with warfarin that were switched to a DOAC, and then calculated the proportion switched to each specific agent (dabigatran, rivaroxaban, apixaban, and edoxaban). Among the patients who were switched, we calculated the proportion who were subsequently switched back to warfarin, to a different DOAC, or had all anticoagulation discontinued. The mean/median number of visits in the PINNACLE dataset and the mean/median time of follow‐up were also obtained. Additionally, patterns were determined after stratifying patients based on CHA_2_DS_2_‐VASc score (groups: score 0‐1, score 2‐3, and score 4 or more). For the population of patients who were switched, demographic, clinical, laboratory, and medication information was compared across groups.

Categorical variables are reported as numbers (percentages) and continuous variables are reported as medians (25th and 75th percentiles) or means (SD). The significance of observed differences was tested using a Wilcoxon rank sum test or *t*‐test for continuous variables and chi‐square test for categorical variables. To identify significant predictors of switching, a multivariate model was created, including all variables with *P* < .1 in bivariate testing.

The multivariate analyses of clinical variables were modeled in different ways: (a) each component of the CHA_2_DS_2_‐VASc individually, (b) CH_2_ADS_2_‐VASc modeled per 1‐point increase along with noncomponent of the CHA_2_DS_2_‐VASc covariates, (c) CHA_2_DS_2_‐VASc modeled as 2‐6 vs 0 or 1 without HAS‐BLED score, and (d) including both CHA_2_DS_2_‐VASc scores and HAS‐BLED scores modeled per 1‐point increase. Since there are co‐linear variables in the risk scores, the CHA_2_DS_2_‐VASc score was modeled both with and without the HAS‐BLED score. Practice‐level analyses were conducted by aggregating patients initially managed with warfarin at the practice‐level. The distribution of the proportion of patients receiving warfarin who were switched to a DOAC was determined across practices. After the distribution was examined, additional practice‐level analyses were conducted by stratifying practices into tertiles based on the proportion of patients who were switched to a DOAC (tertiles were arranged from lowest switching rates to highest).

To measure practice variation, the median odds ratio (MOR) was used. The MOR is able to provide cluster‐level variance when a multilevel regression analysis is performed and can assess variation in a mixed effects model. An MOR >1.2 suggests clinically significant variation in the outcome among a unit after adjustment for fixed effects. In this analysis, an MOR >1.2 suggests clinically significant variation among practices in switching from warfarin to a DOAC.^12^

## 3 RESULTS

### 3.1 Study cohort

**FIGURE 1:**
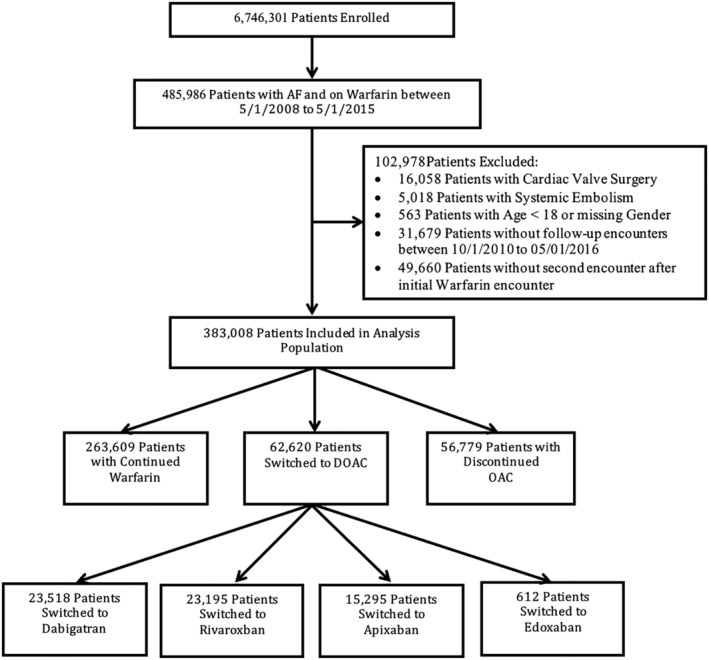
Study population

There were 485 986 patients in the PINNACLE registry with AF on warfarin between May 1, 2008 and May 1, 2015. Of these patients, 102 978 were excluded from the analysis due to cardiac valve surgery, systemic embolism, age less than 18, missing sex, or missing follow up encounters (Figure 1). This resulted in 383 008 patients included in the analysis with at least one prescription for warfarin for AF between May 1, 2008 and May 1, 2015, who were eligible to be switched to a DOAC. Overall demographic, clinical, and practice‐level characteristics of the study population from the PINNACLE registry are shown in Table S1. The mean age of the population was 73.6 (SD 10.7) years, 57.5% were male (n = 220 097), 69.0% were white (n = 264 088), and 3.2% (n = 12 283) were black or African American. Chronic kidney disease (CKD) was seen in 2.2% (n = 8467). Among patients in this analysis, 0.8% (n = 2136) had a glomerular filtration rate (GFR) 12‐29 mL/min and 0.1% (n = 548) a GFR < 15 mL/min. The median CHA_2_DS_2_‐VASc score was 4.0 (IQR 3.0‐5.0) and HAS‐BLED score was 2.0 (IQR 2.0‐3.0). Practices were geographically distributed across the country, with 52.1% in the Southern region and 38.1% in urban locations.

### 3.2 Switching from warfarin to DOAC

Of the 383 008 patients with AF on warfarin, 16.3% (n = 62 620) were switched to a DOAC, while 68.8% (n = 263 609) were continued on warfarin and 14.8% (n = 56 779) had anticoagulation discontinued (Figure 1). Among those who were switched to DOACs, 37.6% (n = 23 518) were switched to dabigatran, 37.0% (n = 23 195) to rivaroxaban, 24.4% (n = 15 295) to apixaban, and 1.0% (n = 612) to edoxaban. The median (25th and 75th percentiles) number of visits from the baseline visit to switching to DOAC was 3.0 (2–5) with mean value of 4.2 (SD 3.6) visits. The median (25th and 75th percentile) duration from the baseline visit to switching to DOAC was 336.0 (118‐767) days with mean duration of 517.0 (SD 530.2) days.

Patterns of switching stratified by CHA_2_DS_2_‐VASc score tertiles are shown in Figure S1. Among patients with a CHA_2_DS_2_‐VASc score of 0‐1 (n = 34 163), 20.7% (n = 7083) were switched to DOAC, 58.8% (n = 20 077) were continued on warfarin, and 20.5% (n = 7003) had anticoagulation discontinued. For patients with a CHA_2_DS_2_‐VASc score of 2‐3 (n = 144 761), 17.1% (n = 24 769) were switched to DOAC, 68.4% (n = 99 064) were continued on warfarin, and 14.5% (n = 20 928) had anticoagulation discontinued. Among patients with a CHA_2_DS_2_‐VASc score of 4 or more (n = 204 084), 15.1% (n = 30 768) were switched to DOAC, 70.8% (n = 144 468) were continued on warfarin, and 14.1% (n = 28 848) had anticoagulation discontinued. Switching among patients with a GFR 15‐29 mL/min was 12.3% (n = 262) and among patients with a GFR < 15 mL/min was 6.4% (n = 35).

### 3.3 Predictors of switching

Baseline demographic, clinical, and practice characteristics of the overall population with AF treated with warfarin (Table S1), patients who were and were not switched (Table S2), and within specific medication groups (Table S3) are shown. Patients switched from warfarin to DOACs tended to be younger, diabetic, have private insurance, and a history of prior stroke; while those who were not switched tended to have coronary artery disease and heart failure (all *P* < .001). The mean CHA_2_DS_2_‐VASc score for those switched was 3.5 (SD 1.7) and for those not switched 3.7 (SD 1.6); the mean HAS‐BLED score for those switched was 2.2 (SD 1.0) and for those not switched was 2.2 (SD 0.9, all *P* ≤ .005).

### 3.4 Multivariate models for predictors of switching to a DOAC

**FIGURE 2:**
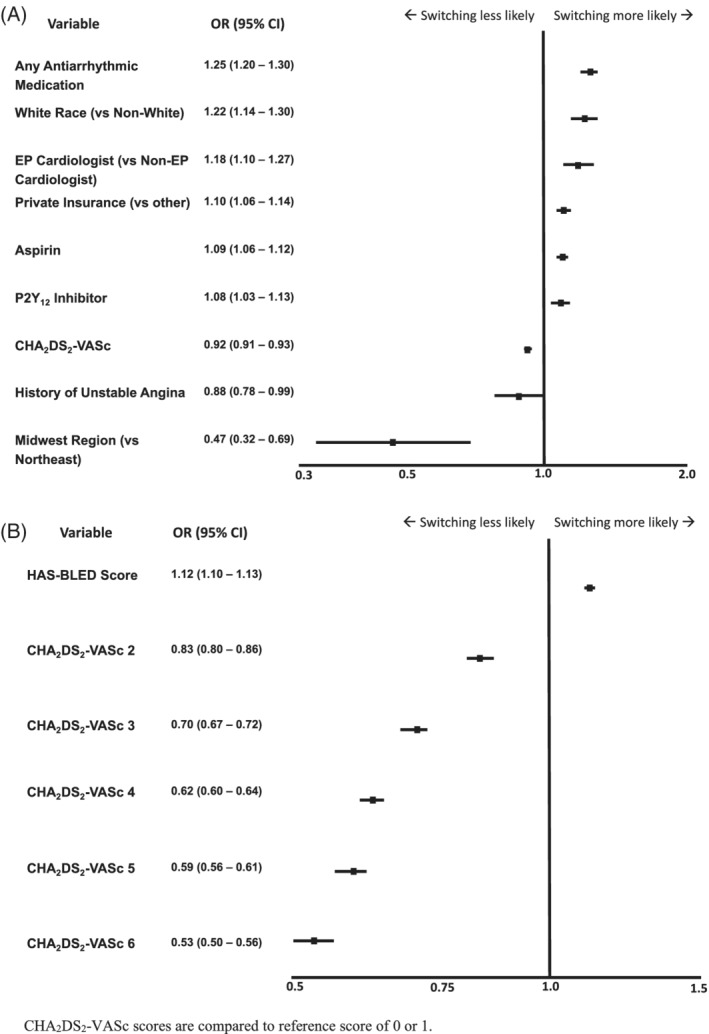
Multivariate hierarchical regression model with, A, CHA_2_DS_2_‐VASc and noncomponent covariates; B, CHA_2_DS_2_‐VASc and HAS‐BLED scores (CHA_2_DS_2_‐VASc scores are compared to reference score of 0 or 1). Variables with nonsignificant associations included History of Stable Angina (OR 1.05, 95%CI 0.99‐1.11), Ethnicity: Hispanic or Latino (vs Not) (OR 1.03 95%CI 0.89‐1.19), Current or Former Smoker (vs Never Smoker) (OR 1.01 95%CI 0.98‐1.04), Other (vs Nurse Practitioner) (OR 1.00 95%CI 0.84‐1.19), Physician Provider (vs Nurse Practitioner) (OR 1.00 95%CI 0.91‐1.10), South Region (vs Northeast) (OR 0.72 95%CI 0.48‐1.10), and West Region (vs Northeast) (OR 0.91 95%CI 0.65‐1.28)

A multivariate hierarchal regression model with the CHA_2_DS_2_‐VASc score and noncomponent covariates (Figure 2a) showed that switching to DOACs was significantly more likely among patients of white race (OR, 1.22; 95%CI, 1.14‐1.30), with private insurance (OR, 1.10; 95%CI, 1.06‐1.14), and those cared for by an electrophysiologist (OR, 1.18; 95%CI, 1.10‐1.27). Switching was significantly less likely per 1‐point increase in CHA_2_DS_2_‐VASc (OR, 0.92; 95%CI, 0.91, 0.93) and at practices in the Midwest as compared with the Northeast region (OR, 0.47; 95%CI, 0.32‐0.69). A multivariate hierarchical regression model comparing CHA_2_DS_2_‐VASc scores to scores of 0‐1 showed that the odds of switching decreased as the CHA_2_DS_2_‐VASc score increased when modeled both with and without the HAS‐BLED score (Figures 2b and S2). Patients with higher HAS‐BLED scores, per 1‐point increase, showed more propensity to switch (OR, 1.12; 95%CI 1.10‐1.13).

The findings were similar when the multivariate hierarchal regression model included clinical covariates of the CHA_2_DS_2_‐VASc score (Figure S3). Specifically, switching to DOACs was more likely with white race vs non‐white (OR, 1.38; 95%CI, 1.29‐1.47), private insurance vs other (OR, 1.08; 95%CI, 1.04‐1.12), prior stroke/transient ischemic attack (TIA) (OR, 1.05; 95%CI, 1.01‐1.10), and electrophysiologists vs nonelectrophysiologist cardiologist (OR, 1.16; 95%CI, 1.08‐1.24). Switching was less likely with age per 10‐year increase (OR, 0.82; 95%CI, 0.81‐0.83), sex: male vs female (OR, 0.92; 95% CI, 0.90‐0.95), history of heart failure (OR, 0.85; 95%CI, 0.82‐0.88), history of diabetes (OR, 0.97; 95%CI, 0.94‐1.00), and Midwest vs Northeast Region (OR, 0.47; 95%CI, 0.32‐0.68).

### 3.5 Multiple switching

**FIGURE 3:**
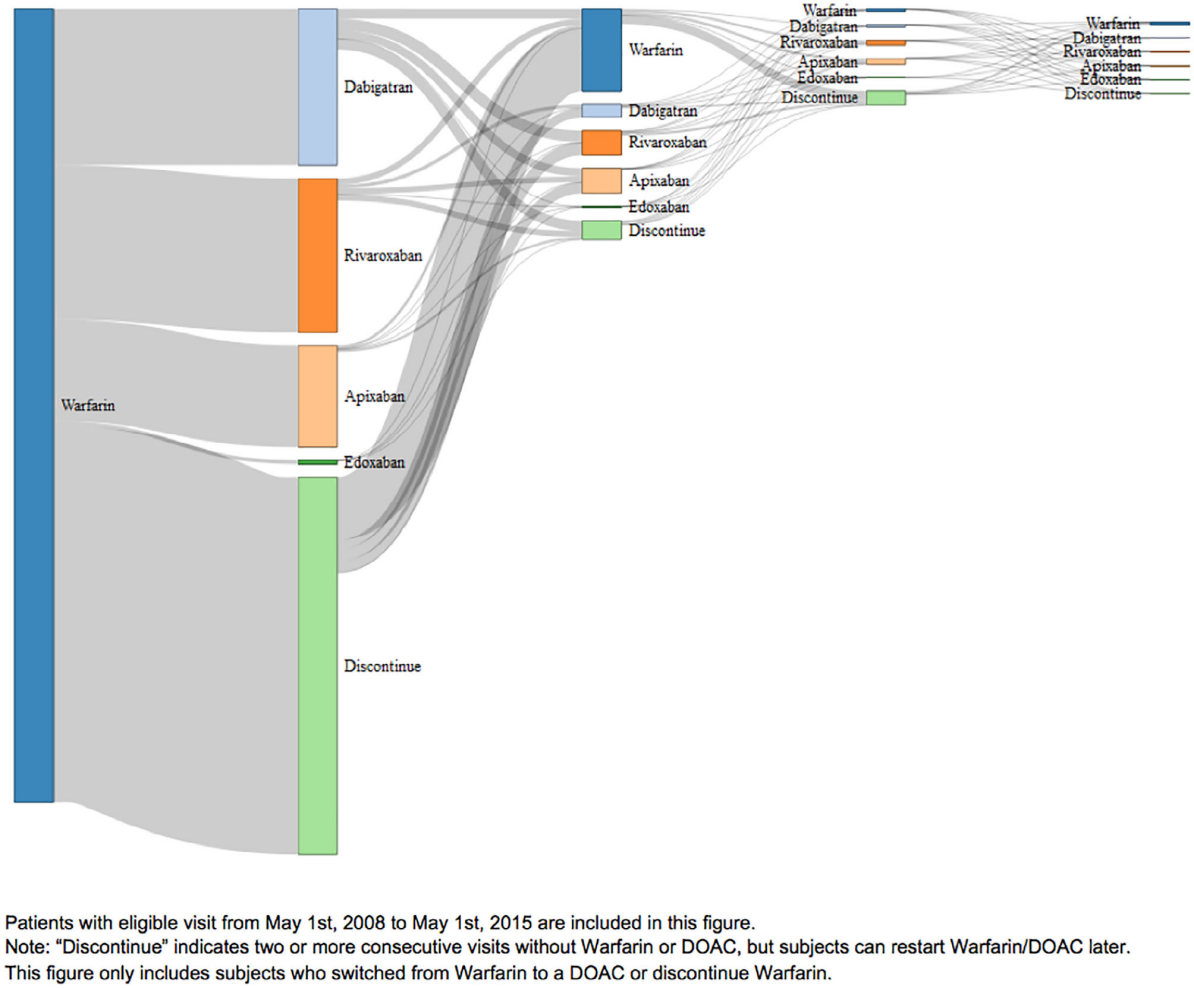
Sankey diagram of multiple anticoagulant switching

Among the 62 620 patients switched to a DOAC, 4.7% (n = 2960) were switched back to warfarin, 7.5% (n = 4692) were switched to another type of DOAC, and 4.6% (n = 2880) eventually discontinued all forms of anticoagulation (Figure S4). A visual representation of multiple changes in anticoagulation is shown in a flow cart with the width of each connection proportional to the number of patients switched (ie, a Sankey diagram; Figure 3).

### 3.6 Practice variation in switching

**FIGURE 4:**
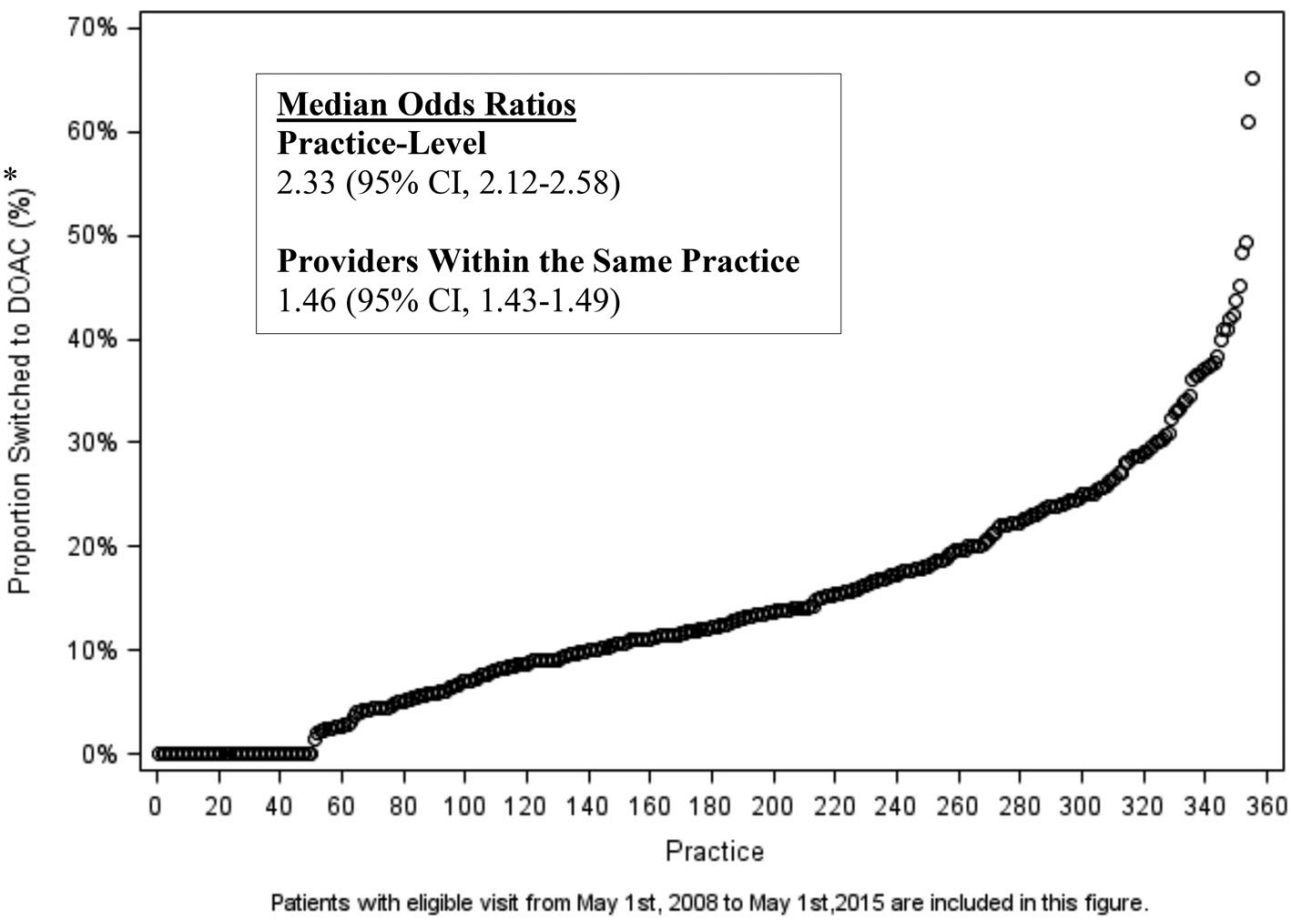
Practice variation for patients switched to DOAC. *Proportion of patients switched at each of the 355 participating centers

There was substantial variation in switching patterns at the practice‐level (MOR, 2.31; 95%CI, 2.11‐2.56) and for providers within the same practice (MOR, 1.46; 95%CI, 1.43‐1.49). A graphical representation of practice variation in switching to DOACs among 355 practices (each practice is represented by a number on the x‐axis, arranged with increasing numbers representing a larger proportion of patients switched in that practice) is shown in Figure 4.

The 355 practices were divided into tertiles (arranged from lowest switching rates to highest) based on the proportion of individuals switched from warfarin to DOACs (Table S4), with tertile 1 comprised practices with the lowest switching rates. Practices with higher switching rates tended to have higher clinical volume, more electrophysiologists, and were more likely to be located in the West region. Tertile 1 was comprised of 36.8% urban locations with mean (SD) visits per year of 11 562 (SD 18 619), 84.9% physician providers, and 5.9% electrophysiologists. Tertile 2 was comprised 45.0% urban locations, mean (SD) visits per year of 18 259 (SD 17 351), 83.2% physician providers, and 8.2% electrophysiologists. Tertile 3 was comprised 33.9% urban locations, mean (SD) visits per year 22 253 (SD 27 024), 84.5% physician providers, and 9.6% electrophysiologists.

### 3.7 Timing of switching

As time progressed, the proportion of individuals switched from warfarin to DOACs increased (Table S5). Among patients switched, 13.8% (n = 8639) were switched from May 2010 to December 2011, 34.2% (n = 62 620) from January 2012 to December 2013, and 52.0% (n = 32 566) from January 2014 to April 2016. Choice of agent varied, with the majority of patients who were switched early receiving dabigatran, but rivaroxaban and apixaban became the predominant agents for later time periods in the analysis. The majority of switches occurred more than 1 year after the index visit (Table S6), with 47.2% (n = 29 560) >365 days after the first visit.

## 4 DISCUSSION

This analysis from a large, national outpatient registry demonstrates that approximately 1 in 6 AF patients treated with warfarin, who were eligible to be switched to a DOAC, were switched between October 1, 2010 and May 1, 2016. There were significant differences in switching based on clinical/sociodemographic factors, and substantial variation in care patterns across practices. This relatively low rate of switching represents an important target for improvement as DOACs have been established as first line therapy for most patients with nonvalvular AF.^4^

The updated ACC/AHA/HRS guidelines recommend DOAC therapy over warfarin given data demonstrating comparable efficacy with less bleeding. A meta‐analysis of randomized controlled trials demonstrated that DOACs provide significant reduction in stroke, intracranial hemorrhage, and mortality with similar major bleeding compared to warfarin.^13^ Against this backdrop, previous analyses from the PINNACLE registry have shown higher rates of DOAC initiation in AF than the switching we observed, with approximately 60% of individuals newly initiated on anticoagulation receiving a DOAC.^5^, ^6^, ^7^, ^8^, ^9^

Potential explanations for the relatively low rate of switching include therapeutic inertia, where providers may not choose to make a change to anticoagulation therapy if a patient has been stable on warfarin without adverse events. Additionally, when DOACs were new, some providers may have waited for accumulating evidence of safety and benefit prior to changing therapy. This may explain why more switching occurred later in the study. Providers may have also chosen to not switch patients they believed had adequate Time in Therapeutic Range (TTR) on warfarin. European guidelines suggest that providers can consider continuing warfarin if patients are able to maintain a TTR on warfarin ≥70% of the time.^14^, ^15^ However, studies suggest that less than 50% of patients on warfarin are able to maintain a TTR ≥70% at 6 months and a smaller proportion of these patients are able to maintain a TTR ≥70% at 12 months.^16^ Finally, cost is a significant barrier to DOAC use for many patients in the United States, but is less of an issue in Europe where there is widespread prescription drug coverage. This is further emphasized by a study from a Danish cohort that showed significantly higher rates of switching than we observed in our analysis.^17^

Broadly speaking, the substantial practice level variation suggests that there are multiple mechanisms underlying these findings. Both at the practice level and among providers within the same practice, there was substantial variation in switching patterns, emphasizing a lack of a unified approach to switching patients from warfarin to DOACs. With some practices switching approximately 65% of patients, while others did not switch any, this highlights the need for additional performance improvement efforts to better align care with practice guidelines.

Another key finding from our analysis is that switching was less prevalent among patients with increasing age, non‐white race, those without private insurance, and men. There have been numerous disparities in many aspects of AF management, including initiation of DOACs and ablation.^5^, ^6^, ^7^, ^8^, ^9^ Although predictors of switching have not been extensively examined in the literature, disparities in DOAC initiation have been observed based on age, race, insurance status, geographic region, female sex, lower household income, higher stroke risk, and higher bleeding risk.^18^, ^19^ In a Danish study that specifically focused on switching from warfarin to DOACs, it was similarly observed that younger age, female sex, history of stroke, and history of bleeding were predictors of switching.^17^ Our analysis adds to this literature and further heightens the need for quality improvement efforts to address and close these disparities.

It is also notable that switching was more prevalent with increased bleeding risk (based on HAS‐BLED scores) but less prevalent with increased stroke risk (based on CHA_2_DS_2_‐VASc scores). We know from other studies that bleeding avoidance is an important driver of decision‐making^20^ and represents an important assessment and reassessment of anticoagulation choices. Providers may have been cautious to switch patients at higher risk of stroke because warfarin was the established agent and because of the inertia to change in the absence of a specific clinical issue. Conversely, the ability to lower bleeding risk with a specific therapeutic intervention may represent a key element in decision‐making. The higher likelihood of switching per 1‐point increase in HAS‐BLED may be related to the lower bleeding risk of DOAC therapy compared to warfarin.^21^, ^22^, ^23^

The lower likelihood of switching among individuals per 1‐point increase in the CHA_2_DS_2_‐VASc score differs from previous registry data on DOAC initiation. Numerous studies have shown that DOAC initiation was less likely with both increasing stroke and bleeding risk.^6^, ^7^, ^14^ This was believed to be related to the lack of commercially available bleeding reversal agents and commonalities among components of the CHA_2_DS_2_‐VASc and HAS‐BLED scores, meaning a higher stroke risk correlates with a higher bleeding risk. Although reversal agents for DOACs are now available, these were not in wide use during the majority of our analysis. As DOAC reversal agents become more widely available, providers may be more likely to switch patients in the future.

Another significant predictor of switching was treatment by an electrophysiologist. This observation may reflect either lack of comfort with these newer agents or decreased awareness of potential benefits among nonsubspecialty practitioners. As a result, referrals to electrophysiologists would impact which patients would be switched. Lower rates of referrals to specialists have been reported in patients post‐MI among different racial and lower socioeconomic groups,^24^ and if such disparities are also present among patients with AF, this would in turn affect switching to DOACs.

The relatively low rate of switching and the potential differences observed in the PINNACLE registry may serve as an important performance improvement target for treatment of patients with AF. Switching patients from warfarin to DOACs is especially important given the 2019 update to the ACC/AHA/HRS AF guidelines, which favor DOACs over warfarin for stroke prevention with a class 1A recommendation.^4^ Increased cost and other barriers to switching must be addressed, including patient perspectives and preferences. The large degree of practice variation seen in this study highlights the lack of a standardized approach for switching patients.

### 4.1 Limitations

There were a few important limitations of this analysis. First, since the PINNACLE registry contains data abstracted from practices participating in a quality improvement registry, it is possible that the data may not be representative of other practices in the United States. This program provides additional support to facilitate guideline recommended treatments that may not be available in most practices. Information such as accessibility to INR testing (ie, patients living far away from testing centers), household income, and drug benefit plans would be helpful to better define the role of cost in limiting switching. Additionally, as with any clinical management decision, there are often relevant factors that fall outside of information that can be collected in a registry, such as patient preference and unmeasured practice variation. Other factors such as nonadherence, or TTR with warfarin may have affected anticoagulation patterns and switching to DOACs, but were not collected in the PINNACLE registry. Another limitation is the relatively sparse data on renal function available for our analysis. Renal dysfunction is an important consideration in choosing anticoagulation for AF patients and previously was a limitation for DOAC use, although current guidelines now suggest it is reasonable to anticoagulate these patients with apixaban.^4^ With a relatively low proportion of patients with CKD, we were not able to systematically examine the association of renal function on switching.

## 5 CONCLUSIONS

In a contemporary cohort of patients with AF treated with warfarin, switching from warfarin to DOACs was relatively uncommon. Switching was less likely with rising stroke risk, but more likely with higher bleeding risk. Additionally, there was significant practice variation in switching. In the context of the 2019 update to the ACC/AHA/HRS guidelines, which favor DOACs over warfarin, these findings may help benchmark performance and identify areas of improvement.

## CONFLICT OF INTEREST

The authors declare no potential conflict of interests.

## AUTHOR CONTRIBUTIONS

Salim S. Virani: American College of Cardiology (Associate Editor for Innovations, acc.org). James V. Freeman: Research Support: ACC NCDR; Modest consulting/advisory board: Medtronic, Boston Scientific, Biosense Webster, Janssen Pharmaceuticals. Nihar R. Desai: Research Support: Centers for Medicare and Medicaid Services; Consulting/advisory board: Amgen, Astra Zeneca, Boehringer Ingelheim, Relypsa. All other authors have no disclosures at this time.

## Supporting information


**Figure S1** Switching from Warfarin to DOAC Stratified by CHA_2_DS_2_‐VASc Score Tertiles

**Figure S2** Univariate Hierarchical Regression Model with CHA_2_DS_2_‐VASc Score Only

**Figure S3** Predictors of Switching from Warfarin to DOACs (with Covariates of the CHA_2_DS_2_‐VASc Score)

**Figure S4** Multiple Anticoagulation Switches Flow Chart

**Table S1** Patient Characteristics, Entire Population Initially Managed on Warfarin

**Table S2** Patient Characteristics ‐ Switched to DOAC vs Not Switched

**Table S3** Patients Characteristics ‐ by Switching Cohorts

**Table S4** Practice Characteristics ‐ All Analysis Population

**Table S5** Switching from Warfarin to DOAC by Time Period

**Table S6** Time from Initial Visit to First Switch
