# Supplementary material for: Switching warfarin to direct oral anticoagulants in atrial fibrillation: Insights from the NCDR PINNACLE registry
Source: Clin Cardiol. 2020 May 6;43(7):743–51. doi: 10.1002/clc.23376 (PMC7368350; doi:10.1002/clc.23376)

**Supplemental Figure 1:** Switching from Warfarin to DOAC Stratified by $CHA_2DS_2$-VASc Score Tertiles

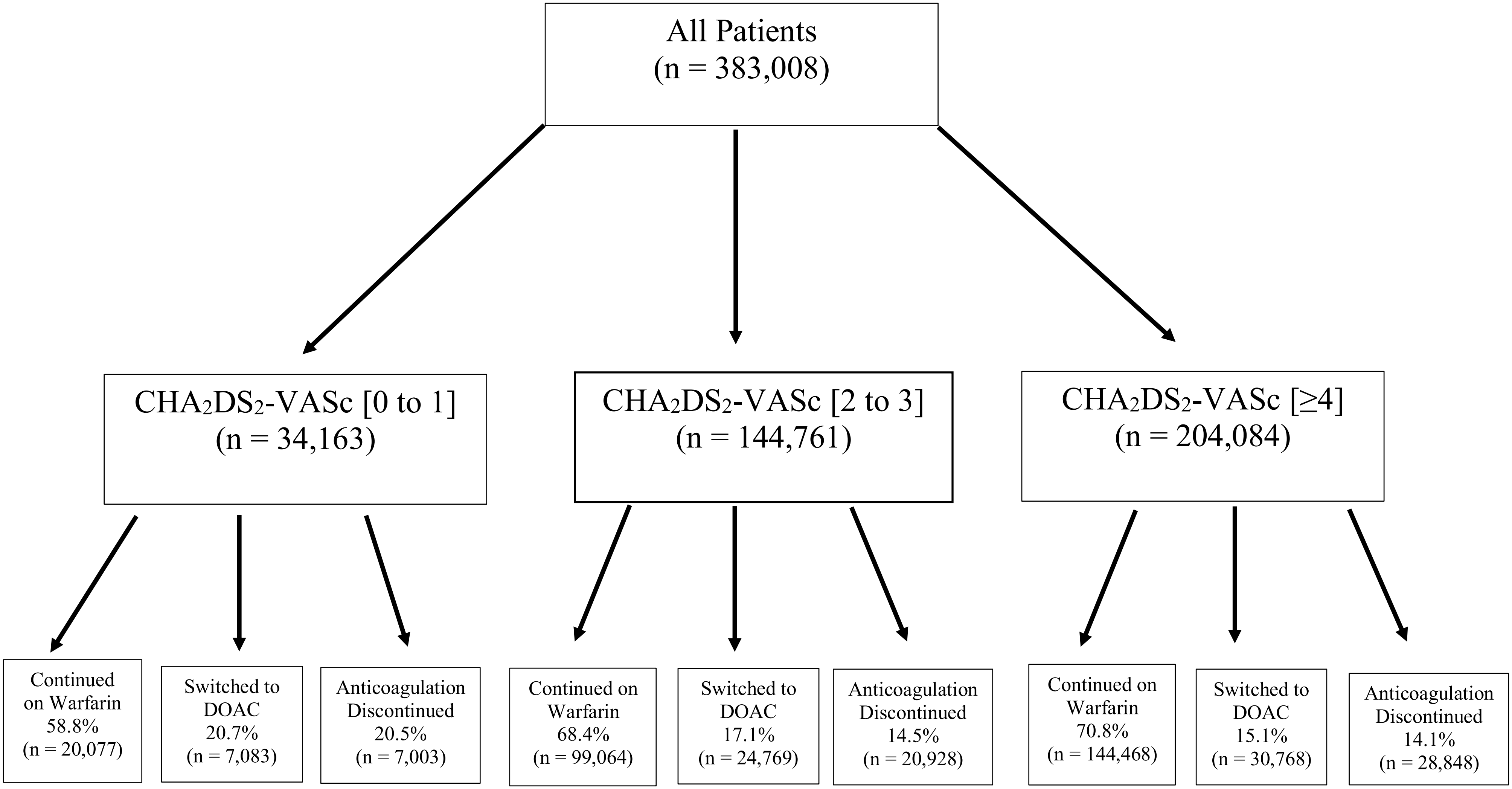

Supplement: Supplementary file 1 — Figure S1 Switching from Warfarin to DOAC Stratified by CHA2DS2‐VASc Score Tertiles [file CLC-43-743-s001.pdf]
