# Supplementary material for: Switching warfarin to direct oral anticoagulants in atrial fibrillation: Insights from the NCDR PINNACLE registry
Source: Clin Cardiol. 2020 May 6;43(7):743–51. doi: 10.1002/clc.23376 (PMC7368350; doi:10.1002/clc.23376)

Supplemental Figure 2: Univariate Hierarchical Regression Model with CHA_2DS_2-VASc Score Only

Variable	OR (95% CI)
CHA_2DS_2-VASc 2	0.90 (0.86 – 0.93)
CHA_2DS_2-VASc 3	0.79 (0.76 – 0.81)
CHA_2DS_2-VASc 4	0.72 (0.70 – 0.75)
CHA_2DS_2-VASc 5	0.71 (0.68 – 0.73)
CHA_2DS_2-VASc 6	0.68 (0.65 – 0.71)

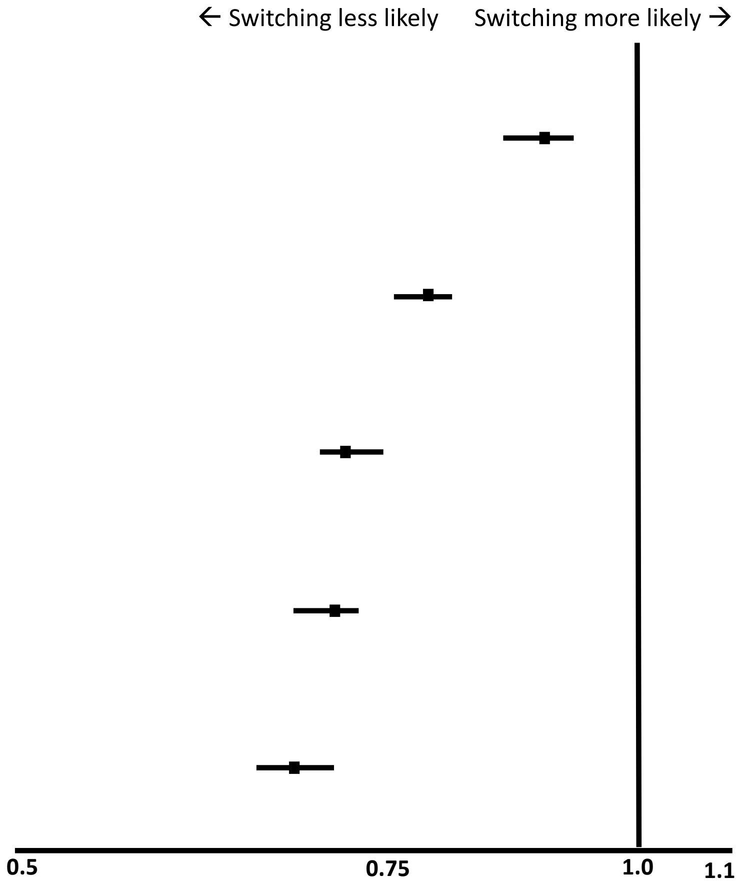

Supplement: Supplementary file 2 — Figure S2 Univariate Hierarchical Regression Model with CHA2DS2‐VASc Score Only [file CLC-43-743-s002.pdf]
