# Supplementary material for: Switching warfarin to direct oral anticoagulants in atrial fibrillation: Insights from the NCDR PINNACLE registry
Source: Clin Cardiol. 2020 May 6;43(7):743–51. doi: 10.1002/clc.23376 (PMC7368350; doi:10.1002/clc.23376)
Supplement: Supplementary file 3 — Figure S3 Predictors of Switching from Warfarin to DOACs (with Covariates of the CHA2DS2‐VASc Score) [file CLC-43-743-s003.pdf]

Supplemental Figure 3: Predictors of Switching from Warfarin to DOACs (with Covariates of the CHA_2DS_2-VASc Score)

Variable	OR (95% CI)
White Race (vs Non-White)	1.38 (1.29 – 1.47)
Any Antiarrhythmic Medication	1.23 (1.18 – 1.28)
EP Cardiologist*	1.16 (1.08 – 1.24)
History of Hypertension	1.14 (1.10 – 1.18)
History of Stable Angina	1.12 (1.06 – 1.19)
Aspirin	1.12 (1.08 – 1.15)
$P2Y_{12}$ Inhibitor	1.10 (1.05 – 1.16)
Private Insurance (vs other)	1.08 (1.04 – 1.12)
History of Stroke or TIA	1.05 (1.01 – 1.10)
Current or Former Smoker	1.03 (1.00 – 1.06)
History of DM	0.97 (0.94 – 1.00)
History of PAD	0.96 (0.91 – 1.00)
Male Sex (vs Female)	0.92 (0.90 – 0.95)
History of CAD	0.87 (0.84 – 0.91)
History of CABG	0.87 (0.83 – 0.92)
History of heart failure	0.85 (0.82 – 0.88)
Age (per 10 year increase)	0.81 (0.82 – 0.83)
Midwest Region (vs Northeast)	0.47 (0.32 – 0.68)

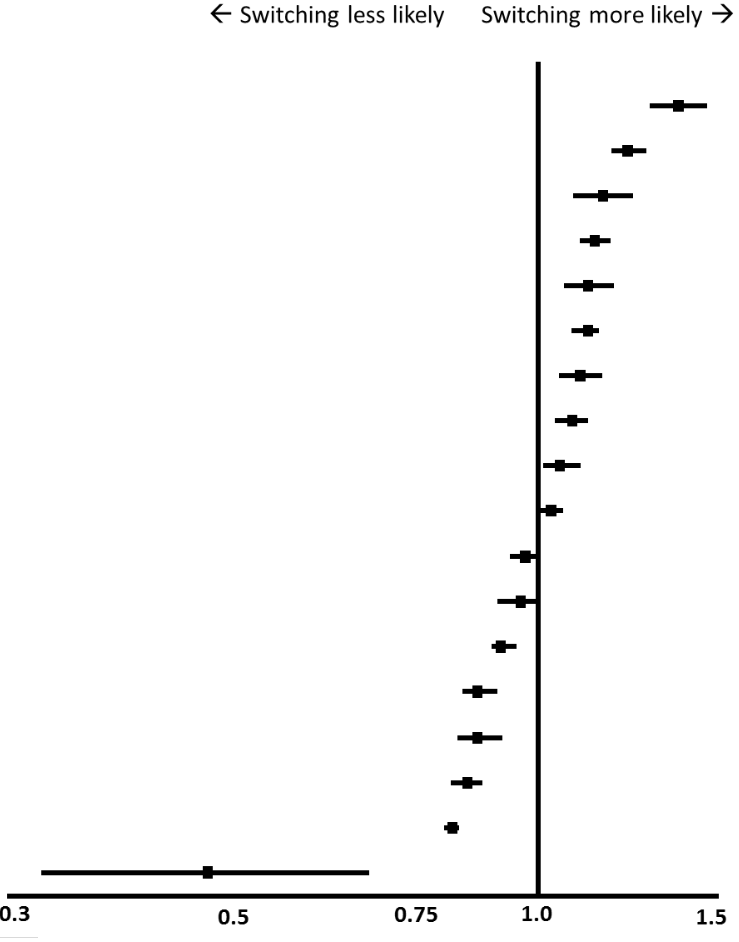

* vs non-EP Cardiologist

Variables with non-significant associations included History of PCI (OR 1.02, 95%CI 0.97 – 1.07), Physician Provider (vs. Nurse Practitioner) (OR 1.00 95%CI 0.91 – 1.10), Other (vs. Nurse Practitioner) (OR 0.99 (95%CI 0.84 – 1.18), Ethnicity: Hispanic or Latino (vs. Not) (OR 0.98, 95%CI 0.85 – 1.14), History of MI (OR 0.98, 95%CI 0.93 – 1.03), West Region (vs. Northeast) (OR 0.90, 95%CI 0.65 – 1.27), History of Unstable Angina (OR 0.90, 95%CI 0.79 – 1.01), South Region (vs. Northeast) (OR 0.73, 95%CI 0.48 – 1.11)
