# Supplementary figures and images for: Switching warfarin to direct oral anticoagulants in atrial fibrillation: Insights from the NCDR PINNACLE registry
Source: Clin Cardiol. 2020 May 6;43(7):743–51. doi: 10.1002/clc.23376 (PMC7368350; doi:10.1002/clc.23376)

**Supplemental Figure 4: Multiple Anticoagulation Switches Flow Chart**

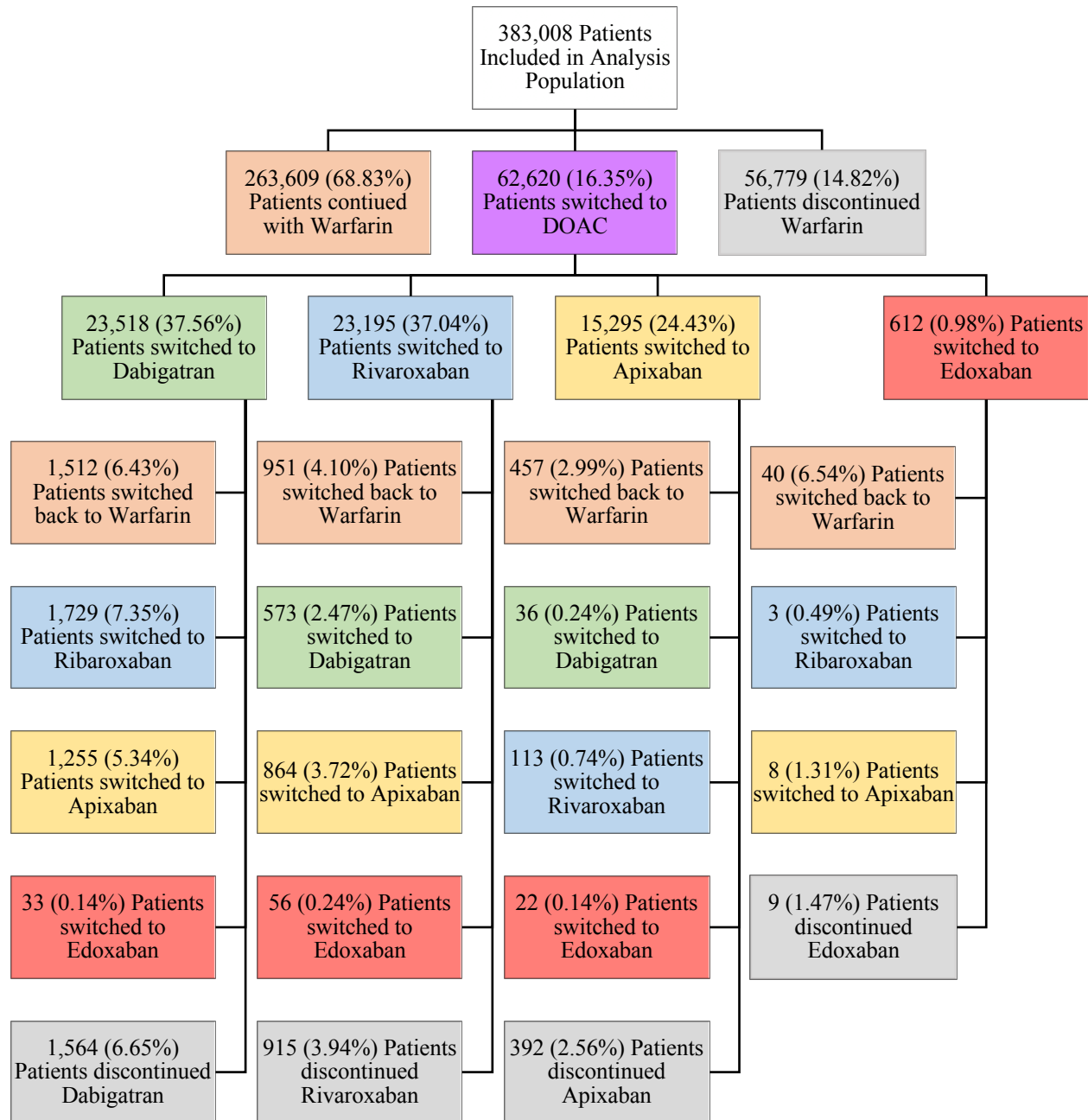

Supplement: Supplementary file 4 — Figure S4 Multiple Anticoagulation Switches Flow Chart [file CLC-43-743-s004.pdf]
