# Supplementary material for: Switching warfarin to direct oral anticoagulants in atrial fibrillation: Insights from the NCDR PINNACLE registry
Source: Clin Cardiol. 2020 May 6;43(7):743–51. doi: 10.1002/clc.23376 (PMC7368350; doi:10.1002/clc.23376)
Supplement: Supplementary file 5 — Table S1 Patient Characteristics, Entire Population Initially Managed on Warfarin [file CLC-43-743-s005.pdf]

Supplemental Table 1: Patient Characteristics, Entire Population Initially Managed on Warfarin

Characteristics	All Patients (N = 383,008)
**Age**	
18 - 50	2.8% (10,803)
51 - 60	8.8% (33,737)
61 - 70	23.3% (89,132)
71 - 80	36.4% (139,358)
> 80	28.7% (109,978)
**Sex**	
Male	57.5% (220,097)
Female	42.5% (162,911)
**Race**	
Missing	27.6% (105,594)
White	69.0% (264,088)
Black	3.2% (12,283)
Other	0.3% (1,043)
**Hispanic or Latino Ethnicity**	
Yes	2.1% (8,050)
No	97.9% (374,958)
**Insurance Type**	
Missing	26.7% (102,442)
None	2.2% (8,457)
Private	47.6% (182,473)
Medicare	22.6% (86,495)
Medicaid	0.4% (1,615)
Other	0.4% (1,526)
**Tobacco Use**	
Never	37.0% (141,632)
Current	10.7% (41,034)
Quit within past 12 months	1.9% (7,320)
Quit more than 12 months ago	34.4% (131,728)
Tobacco screening not performed for medical reasons	0.0% (7)
**Alcohol Use**	
None	13.7% (52,313)
One or fewer alcoholic drinks per week	2.9% (11,103)
2 to 7 alcoholic drinks per week	0.5% (1,883)
8 to 14 alcoholic drinks per week	0.1% (568)

Characteristics	All Patients (N = 383,008)
15 or more alcoholic drinks per week	0.1% (366)
**Comorbidities**	
Hypertension (%)	77.8% (297,790)
Coronary artery disease (%)	49.4% (189,107)
Unstable angina (%)	1.7% (6,637)
Stable angina (%)	8.0% (30,725)
Dyslipidemia (%)	62.6% (239,694)
Congestive heart failure (%)	30.1% (115,348)
Prior stroke or transient ischemic attack (%)	12.6% (48,147)
Prior systemic embolism (%)	0.0% (0)
Peripheral arterial disease (%)	9.4% (35,892)
Diabetes mellitus (%)	23.8% (91,260)
Prior myocardial infarction (%)	14.7% (56,297)
Prior coronary artery bypass graft (%)	9.0% (34,488)
Prior percutaneous coronary intervention (%)	9.0% (34,408)
**Atrial Fibrillation/Flutter Duration**	
First diagnosed	5.9% (22,543)
Paroxysmal	19.1% (73,167)
Persistent	4.1% (15,695)
Long-standing persistent	0.0% (0)
Permanent	0.8% (2,935)
**Left Ventricular Ejection Fraction**	
Missing	53.5% (204,853)
> 70	2.3% (8,663)
50 - 70	31.3% (119,966)
40 - 49	6.0% (22,839)
30 - 39	3.7% (14,310)
< 30	3.2% (12,377)
**Lab Results**	
Hemoglobin A1c (%)	
Mean± SD (N)	8.9±3.5 (5,985)
Total cholesterol (mg/dL)	
Mean± SD (N)	160.2±40.0 (101,641)
High-density lipoprotein cholesterol (mg/dL)	
Mean± SD (N)	47.3±15.5 (98,851)

Characteristics	All Patients (N = 383,008)
Low-density lipoprotein cholesterol (mg/dL)	
Mean± SD (N)	87.8±35.2 (102,163)
International normalized ratio	
Mean± SD (N)	2.4±2.0 (90,106)
**Renal Function Determined by Glomerular Filtration Rate**	
≥ 90	1.4% (5,356)
60 - 89	5.8% (22,079)
30 - 59	5.3% (20,148)
15 - 29	0.6% (2,136)
< 15	0.1% (548)
**Risk Score**	
CHADS2	
Missing	0.0% (0)
Mean± SD (N)	2.1±1.2
Median (Q1, Q3)	2.0 (1.0, 3.0)
Range (Min, Max)	(0.0, 6.0)
CHA_2DS_2-VASc	
Missing	0.0% (0)
Mean± SD (N)	3.7±1.6
Median (Q1, Q3)	4.0 (3.0, 5.0)
Range (Min, Max)	(0.0, 9.0)
CHA_2DS_2-VASc Tertiles	
Score 0 - 1	8.9% (34,163)
Score 2 - 3	37.8% (144,761)
Score 4 or more	53.3% (204,084)
**Bleeding Risk Score**	
HAS-BLED	
Missing	0.0% (0)
Mean± SD (N)	2.2±0.9
Median (Q1, Q3)	2.0 (2.0, 3.0)
Range (Min, Max)	(0.0, 7.0)
**Medications**	
Aspirin (%)	46.1% (176,622)
P2Y12 inhibitor (%)	8.5% (32,507)
Any antiarrhythmic (%)	16.9% (64,615)
Amiodarone (%)	7.4% (28,162)

Characteristics	All Patients (N = 383,008)
Dronedarone (%)	1.1% (4,191)
