# Supplementary material for: Switching warfarin to direct oral anticoagulants in atrial fibrillation: Insights from the NCDR PINNACLE registry
Source: Clin Cardiol. 2020 May 6;43(7):743–51. doi: 10.1002/clc.23376 (PMC7368350; doi:10.1002/clc.23376)
Supplement: Supplementary file 6 — Table S2 Patient Characteristics ‐ Switched to DOAC vs Not Switched [file CLC-43-743-s006.pdf]

Supplemental Table 2: Patient Characteristics - Switched to DOAC vs. Not Switched

Characteristics	Patients switched to DOAC (N = 62,620)	Patients not switched (N = 320,388)	P-value
**Age**			
Mean ± SD (N)	71.7±10.5	74.4±10.3	
≤ 50	3.3% (2,090)	2.7% (8,713)	<.001
51 - 60	11.2% (7,005)	8.3% (26,732)	
61 - 70	27.6% (17,283)	22.4% (71,849)	
71 - 80	36.4% (22,786)	36.4% (116,572)	
> 80	21.5% (13,456)	30.1% (96,522)	
**Sex**			0.353
Male	57.6% (36,090)	57.4% (184,007)	
Female	42.4% (26,530)	42.6% (136,381)	
**Race**			<.001
White	66.7% (41,751)	69.4% (222,337)	
Black	3.4% (2,120)	3.2% (10,163)	
Other	0.3% (183)	0.3% (860)	
**Hispanic or Latino Ethnicity**			<.001
Yes	2.4% (1,525)	2.0% (6,525)	
No	97.6% (61,095)	98.0% (313,863)	
**Insurance Type**			<.001
None	1.9% (1,197)	2.3% (7,260)	
Private	51.8% (32,463)	46.8% (150,010)	
Medicare	19.9% (12,482)	23.1% (74,013)	
Medicaid	0.4% (253)	0.4% (1,362)	
Other	0.6% (391)	0.4% (1,135)	
**Tobacco Use**			<.001
Never	36.1% (22,633)	37.1% (118,999)	

Characteristics	Patients switched to DOAC (N = 62,620)	Patients not switched (N = 320,388)	P-value
Current	12.2% (7,659)	10.4% (33,375)	
Quit within past 12 months	2.4% (1,513)	1.8% (5,807)	
Quit more than 12 months ago	32.5% (2,0375)	34.8% (111,353)	
Tobacco screening not performed for medical reasons	0.0% (0)	0.0% (7)	
**<u>Alcohol Use</u>**			<.001
None	13.4% (8,370)	13.7% (43,943)	
One or fewer alcoholic drinks per week	3.2% (2,027)	2.8% (9,076)	
2 to 7 alcoholic drinks per week	0.4% (250)	0.5% (1,633)	
8 to 14 alcoholic drinks per week	0.1% (75)	0.2% (493)	
15 or more alcoholic drinks per week	0.1% (62)	0.1% (304)	
**<u>Comorbidities</u>**			
Hypertension (%)	78.5% (49,150)	77.6% (248,640)	<.001
Coronary artery disease (%)	46.4% (29,074)	49.9% (160,033)	<.001
Unstable angina (%)	1.5% (908)	1.8% (5,729)	<.001
Stable angina (%)	7.4% (4,626)	8.1% (26,099)	<.001
Dyslipidemia (%)	62.5% (39,152)	62.6% (200,542)	0.739
Congestive heart failure (%)	26.5% (16,577)	30.8% (98,771)	<.001
Prior stroke or transient ischemic attack (%)	14.0% (8,758)	12.3% (39,389)	<.001
Prior systemic embolism (%)	0.0% (0)	0.0% (0)	
Peripheral arterial disease (%)	9.0% (5,629)	9.4% (30,263)	<.001
Diabetes mellitus (%)	24.2% (15,123)	23.8% (76,137)	0.038
Prior myocardial infarction (%)	13.9% (8,735)	14.8% (47,562)	<.001
Prior coronary artery bypass graft (%)	7.7% (4,800)	9.3% (29,688)	<.001
Prior percutaneous coronary intervention (%)	9.3% (5,817)	8.9% (28,591)	0.003

Characteristics	Patients switched to DOAC (N = 62,620)	Patients not switched (N = 320,388)	P-value
**Atrial Fibrillation/Flutter Duration**			<.001
First diagnosed	6.0% (3,764)	5.9% (18,779)	
Paroxysmal	19.9% (12,490)	18.9% (60,677)	
Persistent	3.5% (2,187)	4.2% (13,508)	
Long-standing persistent	0.0% (0)	0.0% (0)	
Permanent	0.6% (403)	0.8% (2,532)	
**Left Ventricular Ejection Fraction**			<.001
> 70	2.3% (1,425)	2.3% (7,238)	
50 - 70	30.1% (18,834)	31.6% (101,132)	
40 - 49	5.4% (3,404)	6.1% (19,435)	
30 - 39	3.4% (2,129)	3.8% (12,181)	
< 30	2.6% (1,649)	3.3% (10,728)	
**Laboratory Results**			
Hemoglobin A1c (%)			
Mean± SD (N)	8.2±3.2 (844)	9.1±3.5 (5,141)	<.001
Total cholesterol (mg/dL)			
Mean± SD (N)	161.3±40.0 (16,492)	160.0±40.0 (85,149)	<.001
High-density lipoprotein cholesterol (mg/dL)			
Mean± SD (N)	48.4±15.9 (15,816)	47.1±15.4 (83,035)	<.001
Low-density lipoprotein cholesterol (mg/dL)			
Mean± SD (N)	88.3±36.2 (16,676)	87.7±35.0 (85,487)	0.063
International formalized ratio			
Mean± SD (N)	2.3±1.8 (15,404)	2.4±2.0 (74,702)	<.001
**Renal Function Determined by Glomerular Filtration Rate**			<.001
≥ 90	1.9% (1,214)	1.3% (4,142)	
60 - 89	7.1% (4,425)	5.5% (17,654)	

Characteristics	Patients switched to DOAC (N = 62,620)	Patients not switched (N = 320,388)	P-value
30 - 59	5.5% (3,420)	5.2% (16,728)	
15 - 29	0.4% (262)	0.6% (1,874)	
< 15	0.1% (35)	0.2% (513)	
**Risk Score**			
CHADS2			
Missing	0.0% (0)	0.0% (0)	
Mean± SD (N)	2.0±1.2	2.1±1.2	<.001
Median (Q1, Q3)	2.0 (1.0, 3.0)	2.0 (1.0, 3.0)	
Range (Min, Max)	(0.0, 6.0)	(0.0, 6.0)	
CHA_2DS_2-VASc			
Missing	0.0% (0)	0.0% (0)	
Mean± SD (N)	3.5±1.7	3.7±1.6	<.001
Median (Q1, Q3)	3.0 (2.0, 5.0)	4.0 (3.0, 5.0)	
Range (Min, Max)	(0.0, 9.0)	(0.0, 9.0)	
CHA_2DS_2-VASc score tertiles			
Score 0 - 1	11.3% (7,083)	8.5% (27,080)	<.001
Score 2 - 3	39.6% (24,769)	37.5% (119,992)	
Score 4 or more	49.1% (30,768)	54.1% (173,316)	
**Bleeding Risk Score**			
HAS-BLED			
Missing	0.0% (0)	0.0% (0)	
Mean± SD (N)	2.2±1.0	2.2±0.9	0.005
Median (Q1, Q3)	2.0 (2.0, 3.0)	2.0 (2.0, 3.0)	
Range (Min, Max)	(0.0, 7.0)	(0.0, 7.0)	
**Medications**			
Aspirin (%)	49.9% (31,222)	45.4% (145,400)	<.001
P2Y12 inhibitor (%)	10.7% (6,703)	8.1% (25,804)	<.001
Any antiarrhythmic (%)	22.4% (14,012)	15.8% (50,603)	<.001

Characteristics	Patients switched to DOAC (N = 62,620)	Patients not switched (N = 320,388)	P-value
Amiodarone (%)	10.4% (6,498)	6.8% (21,664)	<.001
Dronedarone (%)	2.2% (1,385)	0.9% (2,806)	<.001
