# Supplementary material for: Switching warfarin to direct oral anticoagulants in atrial fibrillation: Insights from the NCDR PINNACLE registry
Source: Clin Cardiol. 2020 May 6;43(7):743–51. doi: 10.1002/clc.23376 (PMC7368350; doi:10.1002/clc.23376)
Supplement: Supplementary file 7 — Table S3 Patients Characteristics ‐ by Switching Cohorts [file CLC-43-743-s007.pdf]

Supplemental Table 3: Patients Characteristics - by Switching Cohorts

aracteristics	Continued Warfarin (N = 263,609 patients)	Switched to any DOAC (N = 62,620 patients)	Switched to Dabigatran (N = 23,518 patients)	Switched to Rivaroxaban (N= 23,195 patients)	Switched to Apixaban (N = 15,295 patients)	Switched to Edoxaban (N = 612 patients)
**e**						
0	2.3% (6014)	3.3% (2090)	3.4% (797)	3.7% (864)	2.7% (410)	3.1% (19)
- 60	7.7% (20326)	11.2% (7005)	11.9% (2795)	11.5% (2657)	9.7% (1484)	11.3% (69)
- 70	22.0% (57938)	27.6% (17283)	29.6% (6957)	27.1% (6296)	25.3% (3866)	26.8% (164
- 80	37.3% (98231)	36.4% (22786)	36.1% (8483)	36.1% (8362)	37.3% (5712)	37.4% (229
0	30.8% (81100)	21.5% (13456)	19.1% (4486)	21.6% (5016)	25.0% (3823)	21.4% (131
**x**						
le	57.0% (150305)	57.6% (36090)	60.1% (14124/23518)	57.3% (13287)	54.4% (8320)	58.7% (359
male	43.0% (113304)	42.4% (26530)	39.9% (9394/23518)	42.7% (9908)	45.6% (6975)	41.3% (253
**ce**						
ssing	26.9% (70865)	29.6% (18566)	30.0% (7060)	30.2% (7007)	28.4% (4349)	24.5% (150
nite	69.7% (183657)	66.7% (41751)	67.0% (15749)	65.7% (15228)	67.6% (10335)	71.7% (439
ack	3.2% (8340)	3.4% (2120)	2.7% (635)	3.9% (896)	3.7% (568)	3.4% (21)
her	0.3% (747)	0.3% (183)	0.3% (74)	0.3% (64)	0.3% (43)	0.3% (2)
**spanic or Latino hnicity**						
s	2.1% (5502)	2.4% (1525)	1.8% (423)	2.9% (666)	2.7% (420)	2.6% (16)
	97.9% (258107)	97.6% (61095)	98.2% (23095)	97.1% (22529)	97.3% (14875)	97.4% (596
**urance Type**						
ne	2.1% (5572)	1.9% (1197)	2.0% (471)	1.5% (341)	2.5% (375)	1.6% (10)
vate	45.9% (121017)	51.8% (32463)	54.7% (12858)	49.9% (11568)	50.9% (7784)	41.3% (253
edicare	22.1% (58193)	19.9% (12482)	23.9% (5616)	17.7% (4107)	17.4% (2667)	15.0% (92)
edicaid	0.4% (1040)	0.4% (253)	0.5% (111)	0.4% (99)	0.3% (39)	0.7% (4)
her	0.4% (929)	0.6% (391)	0.7% (162)	0.6% (139)	0.6% (87)	0.5% (3)
**bacco Use**						
ver	37.6% (98999)	36.1% (22633)	33.0% (7768)	37.0% (8573)	39.5% (6034)	42.2% (258
rrent	10.7% (28325)	12.2% (7659)	11.7% (2751)	12.9% (2983)	12.1% (1844)	13.2% (81)
it within past 12 nths	1.8% (4739)	2.4% (1513)	2.2% (507)	2.9% (669)	2.2% (332)	0.8% (5)
it more than 12 months o	34.4% (90703)	32.5% (20375)	32.2% (7577)	31.8% (7371)	34.1% (5209)	35.6% (218
bacco screening not formed for medical sons	0.0% (3)	0.0% (0)	0.0% (0)	0.0% (0)	0.0% (0)	0.0% (0)

aracteristics	Continued Warfarin (N = 263,609 patients)	Switched to any DOAC (N = 62,620 patients)	Switched to Dabigatran (N = 23,518 patients)	Switched to Rivaroxaban (N= 23,195 patients)	Switched to Apixaban (N = 15,295 patients)	Switched t Edoxaban (N = 612 patients)
**cohol Use**						
ne	14.5% (38165)	13.4% (8370)	7.8% (1825)	15.4% (3561)	18.6% (2848)	22.2% (136
e or fewer alcoholic nks per week	3.0% (7876)	3.2% (2027)	2.0% (478)	3.8% (877)	4.3% (655)	2.8% (17)
o 7 alcoholic drinks per ek	0.5% (1424)	0.4% (250)	0.2% (40)	0.5% (110)	0.6% (94)	1.0% (6)
o 14 alcoholic drinks week	0.2% (425)	0.1% (75)	0.0% (10)	0.1% (28)	0.2% (34)	0.5% (3)
or more alcoholic nks per week	0.1% (267)	0.1% (62)	0.0% (11)	0.1% (30)	0.1% (20)	0.2% (1)
**morbidities**						
pertension (%)	77.8% (204991)	78.5% (49150)	78.2% (18383)	78.1% (18118)	79.4% (12142)	82.8% (507
ronary artery disease)	49.5% (130461)	46.4% (29074)	46.8% (10998)	45.8% (10629)	46.3% (7088)	58.7% (359
Unstable angina (%)	1.7% (4453)	1.5% (908)	1.1% (254)	1.5% (356)	1.8% (277)	3.4% (21)
Stable angina (%)	7.9% (20893)	7.4% (4626)	6.3% (1472)	7.7% (1777)	8.5% (1294)	13.6% (83)
slipidemia (%)	62.7% (165348)	62.5% (39152)	62.5% (14702)	62.7% (14535)	62.0% (9487)	69.9% (428
ngestive heart failure)	30.8% (81154)	26.5% (16577)	25.1% (5892)	26.1% (6050)	29.0% (4432)	33.2% (203
or Stroke or transient hemic attack (%)	12.3% (32441)	14.0% (8758)	14.4% (3377)	13.5% (3124)	14.2% (2174)	13.6% (83)
or systemic embolism)	0.0% (0)	0.0% (0)	0.0% (0)	0.0% (0)	0.0% (0)	0.0% (0)
ripheral arterial disease)	9.5% (24949)	9.0% (5629)	8.2% (1918)	9.5% (2197)	9.3% (1425)	14.5% (89)
abetes mellitus (%)	24.0% (63188)	24.2% (15123)	24.1% (5659)	24.3% (5638)	23.5% (3589)	38.7% (237
or myocardial arction (%)	14.5% (38219)	13.9% (8735)	15.4% (3633)	13.3% (3080)	12.7% (1937)	13.9% (85)
or coronary artery pass graft (%)	9.0% (23596)	7.7% (4800)	7.1% (1668)	7.6% (1763)	8.6% (1312)	9.3% (57)
or percutaneous onary intervention (%)	8.6% (22626)	9.3% (5817)	9.1% (2144)	9.1% (2114)	9.8% (1496)	10.3% (63)
**rial prillation//Flutter ration**						
st diagnosed	5.0% (13282)	6.0% (3764)	8.0% (1882)	5.1% (1180)	4.4% (675)	4.4% (27)
roxysmal	18.7% (49344)	19.9% (12490)	19.3% (4550)	20.1% (4657)	20.8% (3175)	17.6% (108
rsistent	4.3% (11238)	3.5% (2187)	3.8% (895)	3.2% (746)	3.4% (519)	4.4% (27)
ng-standing persistent	0.0% (0)	0.0% (0)	0.0% (0)	0.0% (0)	0.0% (0)	0.0% (0)

[illegible]aracteristics	Continued Warfarin (N = 263,609 patients)	Switched to any DOAC (N = 62,620 patients)	Switched to Dabigatran (N = 23,518 patients)	Switched to Rivaroxaban (N= 23,195 patients)	Switched to Apixaban (N = 15,295 patients)	Switched t[illegible] Edoxaban (N = 612 patients)
[illegible]rmanent	0.9% (2370)	0.6% (403)	0.3% (67)	0.7% (154)	1.2% (178)	0.7% (4)
**[illegible]ft Ventricular [illegible]ection Fraction**						
[illegible]ssing	53.1% (139970)	56.2% (35179)	60.6% (14254)	55.2% (12795)	51.8% (7919)	34.5% (211[illegible]
[illegible]0	2.2% (5863)	2.3% (1425)	2.2% (514)	2.4% (546)	2.2% (343)	3.6% (22)
[illegible]- 70	31.4% (82711)	30.1% (18834)	27.2% (6392)	30.5% (7076)	33.4% (5109)	42.0% (257[illegible]
[illegible]- 49	6.1% (16092)	5.4% (3404)	4.9% (1146)	5.7% (1316)	5.8% (886)	9.2% (56)
[illegible]- 39	3.8% (10058)	3.4% (2129)	2.9% (688)	3.6% (830)	3.7% (570)	6.7% (41)
[illegible]0	3.4% (8915)	2.6% (1649)	2.2% (524)	2.7% (632)	3.1% (468)	4.1% (25)
**[illegible]b Results**						
[illegible]moglobin A1c (%)						
[illegible]Mean ± SD (N)	9.1±3.5 (4493)	8.2±3.2 (844)	7.7±3.0 (142)	8.2±3.3 (383)	8.3±3.2 (301)	8.0±3.3 (18[illegible]
[illegible]tal cholesterol (mg/dL)						
[illegible]Mean ± SD (N)	159.7±39.7 (69283)	161.3±40.0 (16492)	160.5±38.9 (6273)	162.6±41.1 (5996)	161.0±40.1 (4010)	155.7±41.2 (213)
[illegible]gh-density lipoprotein [illegible]olesterol (mg/dL)						
[illegible]Mean ± SD (N)	47.1±15.4 (67365)	48.4±15.9 (15816)	48.2±15.9 (6093)	48.5±16.0 (5722)	48.6±15.9 (3790)	45.5±14.2 (21[illegible]
[illegible]w-density lipoprotein [illegible]olesterol (LDL) [illegible]g/dL)						
[illegible]Mean ± SD (N)	87.4±34.6 (69542)	88.3±36.2 (16676)	88.1±38.5 (6374)	89.1±34.2 (6053)	87.6±34.0 (4035)	86.0±53.1 (21[illegible]
[illegible]ernational normalized [illegible]io						
[illegible]Mean ± SD (N)	2.4±2.0 (64043)	2.3±1.8 (15404)	2.2±1.1 (4342)	2.3±2.2 (6264)	2.3±1.8 (4587)	2.5±1.0 (21[illegible]
**[illegible]nal Function [illegible]termined by [illegible]omerular Filtration [illegible]te**						
[illegible]ssing	86.3% (227376)	85.1% (53264)	89.9% (21149)	82.7% (19176)	81.5% (12458)	78.6% (481[illegible]
[illegible]0	1.4% (3562)	1.9% (1214)	1.2% (293)	2.5% (584)	2.1% (320)	2.8% (17)
[illegible]- 89	6.0% (15714)	7.1% (4425)	5.0% (1169)	8.3% (1935)	8.2% (1260)	10.0% (61[illegible]
[illegible]- 59	5.7% (14933)	5.5% (3420)	3.6% (845)	6.1% (1405)	7.3% (1123)	7.7% (47)
[illegible]- 29	0.6% (1593)	0.4% (262)	0.2% (55)	0.4% (90)	0.7% (112)	0.8% (5)
[illegible]5	0.2% (431)	0.1% (35)	0.0% (7)	0.0% (5)	0.1% (22)	0.2% (1)
**[illegible]sk Score**						

aracteristics	Continued Warfarin (N = 263,609 patients)	Switched to any DOAC (N = 62,620 patients)	Switched to Dabigatran (N = 23,518 patients)	Switched to Rivaroxaban (N= 23,195 patients)	Switched to Apixaban (N = 15,295 patients)	Switched to Edoxaban (N = 612 patients)
IADS$_2$						
Missing	0.0% (0)	0.0% (0)	0.0% (0)	0.0% (0)	0.0% (0)	0.0% (0)
Mean ± SD (N)	2.1±1.2	2.0±1.2	2.0±1.2	2.0±1.2	2.1±1.2	2.3±1.3
Median (Q1, Q3)	2.0 (1.0, 3.0)	2.0 (1.0, 3.0)	2.0 (1.0, 3.0)	2.0 (1.0, 3.0)	2.0 (1.0, 3.0)	2.0 (1.0, 3.0
Range (Min, Max)	(0.0, 6.0)	(0.0, 6.0)	(0.0, 6.0)	(0.0, 6.0)	(0.0, 6.0)	(0.0, 6.0)
IA$_2$DS$_2$-VASc						
Missing	0.0% (0)	0.0% (0)	0.0% (0)	0.0% (0)	0.0% (0)	0.0% (0)
Mean ± SD (N)	3.7±1.6	3.5±1.7	3.4±1.7	3.5±1.7	3.7±1.6	3.8±1.7
Median (Q1, Q3)	4.0 (3.0, 5.0)	3.0 (2.0, 5.0)	3.0 (2.0, 4.0)	3.0 (2.0, 5.0)	4.0 (3.0, 5.0)	4.0 (3.0, 5.0
Range (Min, Max)	(0.0, 9.0)	(0.0, 9.0)	(0.0, 9.0)	(0.0, 9.0)	(0.0, 9.0)	(0.0, 9.0)
IA$_2$DS$_2$-VASc score tiles						
Score 0 - 1	7.6% (20077)	11.3% (7083)	12.5% (2949)	11.6% (2686)	9.1% (1393)	9.0% (55)
Score 2 - 3	37.6% (99064)	39.6% (24769)	40.4% (9504)	40.1% (9296)	37.6% (5753)	35.3% (216
Score 4 or more	54.8% (144468)	49.1% (30768)	47.0% (11065)	48.3% (11213)	53.3% (8149)	55.7% (341
**eeding Risk Score**						
AS-BLED						
Missing	0.0% (0)	0.0% (0)	0.0% (0)	0.0% (0)	0.0% (0)	0.0% (0)
Mean ± SD (N)	2.2±0.9	2.2±1.0	2.2±1.0	2.2±1.0	2.3±1.0	2.4±1.0
Median (Q1, Q3)	2.0 (2.0, 3.0)	2.0 (2.0, 3.0)	2.0 (2.0, 3.0)	2.0 (2.0, 3.0)	2.0 (2.0, 3.0)	2.0 (2.0, 3.0
Range (Min, Max)	(0.0, 7.0)	(0.0, 7.0)	(0.0, 7.0)	(0.0, 7.0)	(0.0, 7.0)	(0.0, 6.0)
**edications**						
pirin (%)	44.1% (116277)	49.9% (31222)	48.5% (11412)	50.6% (11744)	50.6% (7735)	54.1% (331
Y12 inhibitor (%)	7.7% (20268)	10.7% (6703)	10.0% (2344)	11.2% (2595)	11.2% (1707)	9.3% (57)
y antiarrhythmic (%)	16.9% (44514)	22.4% (14012)	14.8% (3477)	25.8% (5979)	28.6% (4378)	29.1% (178
Amiodarone (%)	7.0% (18402)	10.4% (6498)	6.8% (1606)	12.3% (2855)	12.8% (1961)	12.4% (76)
Dronedarone (%)	0.9% (2340)	2.2% (1385)	1.9% (439)	2.1% (477)	3.0% (461)	1.3% (8)
