# Supplementary material for: Switching warfarin to direct oral anticoagulants in atrial fibrillation: Insights from the NCDR PINNACLE registry
Source: Clin Cardiol. 2020 May 6;43(7):743–51. doi: 10.1002/clc.23376 (PMC7368350; doi:10.1002/clc.23376)
Supplement: Supplementary file 8 — Table S4 Practice Characteristics ‐ All Analysis Population [file CLC-43-743-s008.pdf]

Supplemental Table 4: Practice Characteristics – All Analysis Population

Characteristics	Tertile 1 (Tertile with least switching) (N=118 Practices) (N=410 Locations) (N=1,104 Providers)	Tertile 2 (N=119 Practices) (N=446 Locations) (N=1,524 Providers)	Tertile 3 (Tertile with most switching) (N=118 Practices) (N=580 Locations) (N=1,882 Providers)
**US Region**			
Northeast	15.3% (18/118)	16.0% (19/119)	11.9% (14/118)
Midwest	24.6% (29/118)	18.5% (22/119)	13.6% (16/118)
South	52.5% (62/118)	52.1% (62/119)	51.7% (61/118)
West	7.6% (9/118)	13.4% (16/119)	22.9% (27/118)
**Urban Location**			
Urban location	36.8% (71/193)	45.0% (143/318)	33.9% (158/466)
**Clinic Volume**			
Visits per year			
Missing	0.0% (0/118)	0.0% (0/119)	0.0% (0/118)
Mean ± SD (N)	11,562±18,619 (118)	18,259±17,351 (119)	22,253±27,025 (118)
Median (Q1, Q3)	5,634 (2,535, 12,978)	13,294 (6,040, 24,654)	12,151 (4,746, 27,046)
Range (Min, Max)	(170, 145,970)	(500, 103,582)	(430, 148,296)
**Provider**			
Physician	84.9% (935/1,101)	83.2% (1265/1,520)	84.5% (1,582/1,872)
Nurse practitioner	10.3% (113/1,101)	10.9% (166/1,520)	11.1% (208/1,872)
Other	4.8% (53/1,101)	5.9% (89/1,520)	4.4% (82/1,872)
**Cardiologist**			
Electrophysiologist	5.9% (65/1,103)	8.2% (125/1,523)	9.6% (181/1,882)
Non-electrophysiologist cardiologist	94.1% (1,038/1,103)	91.8% (1,398/1,523)	90.4% (1701/1,882)
