# Supplementary material for: Switching warfarin to direct oral anticoagulants in atrial fibrillation: Insights from the NCDR PINNACLE registry
Source: Clin Cardiol. 2020 May 6;43(7):743–51. doi: 10.1002/clc.23376 (PMC7368350; doi:10.1002/clc.23376)
Supplement: Supplementary file 9 — Table S5 Switching from Warfarin to DOAC by Time Period [file CLC-43-743-s009.pdf]

Supplemental Table 5: Switching from Warfarin to DOAC by Time Period

	**Subjects switched to DOAC (N=62620)**
First switch in May 2010 to December 2011	13.8% (8639)
Switch to Dabigatran	92.3% (7976)
Switch to Rivaroxaban	7.7% (663)
Switch to Apixaban	0
Switch to Edoxaban	0
First switch in January 2012 to December 2013	34.2% (21415)
Switch to Dabigatran	48.8% (10444)
Switch to Rivaroxaban	40.3% (8626)
Switch to Apixaban	10.6% (2262)
Switch to Edoxaban	0.4% (83)
First switch in January 2014 to April 2016	52.0% (32566)
Switch to Dabigatran	15.7% (5098)
Switch to Rivaroxaban	42.7% (13906)
Switch to Apixaban	40.0% (13033)
Switch to Edoxaban	1.6% (529)
