# Supplementary material for: Switching warfarin to direct oral anticoagulants in atrial fibrillation: Insights from the NCDR PINNACLE registry
Source: Clin Cardiol. 2020 May 6;43(7):743–51. doi: 10.1002/clc.23376 (PMC7368350; doi:10.1002/clc.23376)
Supplement: Supplementary file 10 — Table S6 Time from Initial Visit to First Switch [file CLC-43-743-s010.pdf]

Supplemental Table 6: Time from Initial Visit to First Switch

	Subjects switched to DOAC (N=62620)
Days from Index Visit to First Switch to DOAC	
Within 30 days	7.9% (4977/62620)
31-90 days	12.2% (7625/62620)
91-180 days	12.5% (7810/62620)
181-365 days	20.2% (12648/62620)
After 365 days	47.2% (29560)

Baseline visits: January 1, 2008 to May 1, 2015. Follow-up visits: October 1, 2010 and May 1, 2016
